# Supplementary material for: A Global Screen for Assembly State Changes of the Mitotic Proteome by SEC-SWATH-MS
Source: Cell Syst. 2020 Feb 26;10(2):133–155.e6. doi: 10.1016/j.cels.2020.01.001 (PMC7042714; doi:10.1016/j.cels.2020.01.001)

Q9Y3A6 | TMED5_HUMAN | TMED5 CGI−100 UNQ397/PRO733
Monomer MW [kDa]: 26.005 Monomer expected elution fraction: 52

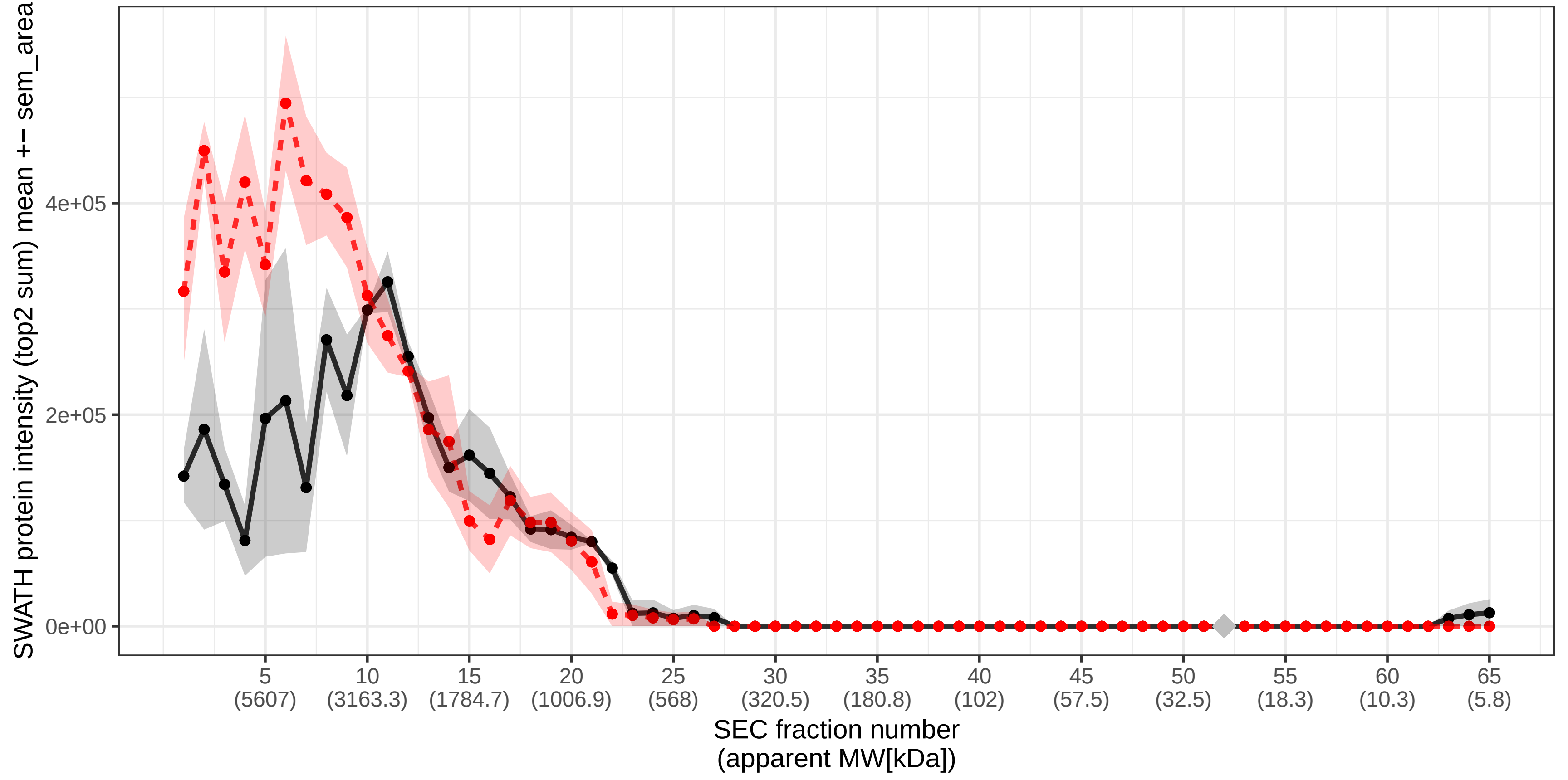

Supplement: Data S1. SEC-SWATH-MS Protein Chromatograms, Related to Figure 1 [file mmc6.zip › SECchrom_Q9Y3A6_TMED5_HUMAN_TMED5_CGI-100_UNQ397_PRO733.pdf]
